# X-Ray Repair Cross Complementing Protein 1 in Base Excision Repair

**DOI:** 10.3390/ijms131217210

**Published:** 2012-12-17

**Authors:** Audun Hanssen-Bauer, Karin Solvang-Garten, Mansour Akbari, Marit Otterlei

**Affiliations:** 1 Department of Cancer Research and Molecular Medicine, Faculty of Medicine, Norwegian University of Science and Technology, N-7489 Trondheim, Norway; E-Mails: audunhb@gmail.com (A.H.-B.); karin.solvang-garten@ntnu.no (K.S.-G.); 2 Department of Cellular and Molecular Medicine, Faculty of Health and Medical Sciences, University of Copenhagen, Blegdamsvej 3B, 2200 N, Denmark; E-Mail: akbari@sund.ku.dk

**Keywords:** BER, XRCC1, disease, DNA damage, polymorphisms, recruitment, repair complex, scaffold

## Abstract

X-ray Repair Cross Complementing protein 1 (XRCC1) acts as a scaffolding protein in the converging base excision repair (BER) and single strand break repair (SSBR) pathways. XRCC1 also interacts with itself and rapidly accumulates at sites of DNA damage. XRCC1 can thus mediate the assembly of large multiprotein DNA repair complexes as well as facilitate the recruitment of DNA repair proteins to sites of DNA damage. Moreover, XRCC1 is present in constitutive DNA repair complexes, some of which associate with the replication machinery. Because of the critical role of XRCC1 in DNA repair, its common variants Arg194Trp, Arg280His and Arg399Gln have been extensively studied. However, the prevalence of these variants varies strongly in different populations, and their functional influence on DNA repair and disease remains elusive. Here we present the current knowledge about the role of XRCC1 and its variants in BER and human disease/cancer.

## 1. X-Ray Repair Cross Complementing Protein 1 (XRCC1)

XRCC1 was identified as a single strand break repair (SSBR) factor because transgene expression of human XRCC1 in the *Xrcc1* deficient Chinese hamster ovary (CHO) cell line EM9 restored its SSBR capacity [1]. The reduced SSBR capacity of EM9 CHO is associated with a tenfold increase in sensitivity to mono-functional alkylating agents, increased sensitivity to X-ray and ultraviolet (UV) irradiation, and increased sister chromatid exchange (SCE). Although XRCC1-deficient cancer cell lines are viable, XRCC1-deficiency in mice is embryonically lethal [2]. The exact physiological role of XRCC1 during foetal development is difficult to determine because XRCC1-deficient embryos die at around the seventh day, but the arrested embryos resemble those from AP-endonuclase 1 (APE1) deficient mice and die at approximately the same stage as DNA ligase 3 (LIG3) deficient mice embryos [3–5]. However, in addition to BER, XRCC1 has also been linked to non-homologous end joining (NHEJ) and possibly nucleotide excision repair (NER), indicating that the underlying causes for the observed embryonic lethality might be complex [6,7]. While full body *Xrcc1* disruption in mice is lethal, Nes-cre conditional *Xrcc1* neuron disruption produces viable offspring. However, the *Xrcc1^Nes–cre^* mice do not survive longer than 4 months, and while they accumulate DNA strand breaks in neurons none developed cancer within their short lifespan. *Xrcc1^Nes–cre^* mice are characterized by delayed growth, smaller brain size, loss of cerebellar interneurons, abnormal hippocampal function, and develop mild ataxia accompanied by episodic seizures [8]. Similar to the XRCC1-deficient CHO cells, cells from XRCC1 deficient mice embryos and *Xrcc1^Nes–cre^* mice neurons show hypersensitivity to mutagens [2,8]. Transgene complementation in *Xrcc1*^−/−^ mice expressing less than 10% of the normal level of XRCC1 is sufficient to rescue the embryonic development and produce healthy fertile adults [9]. However, heterozygous *Xrcc1*^+/−^ mice, expressing 50% of the normal level of XRCC1, show increased precancerous lesions in the colon and liver toxicity upon ingestion of alkylating agents [10]. This suggests that the cellular concentration of XRCC1 and likely the regulation of associated multiprotein complexes are important for genomic maintenance.

## 2. Base Excision Repair (BER)

**Figure 1:**
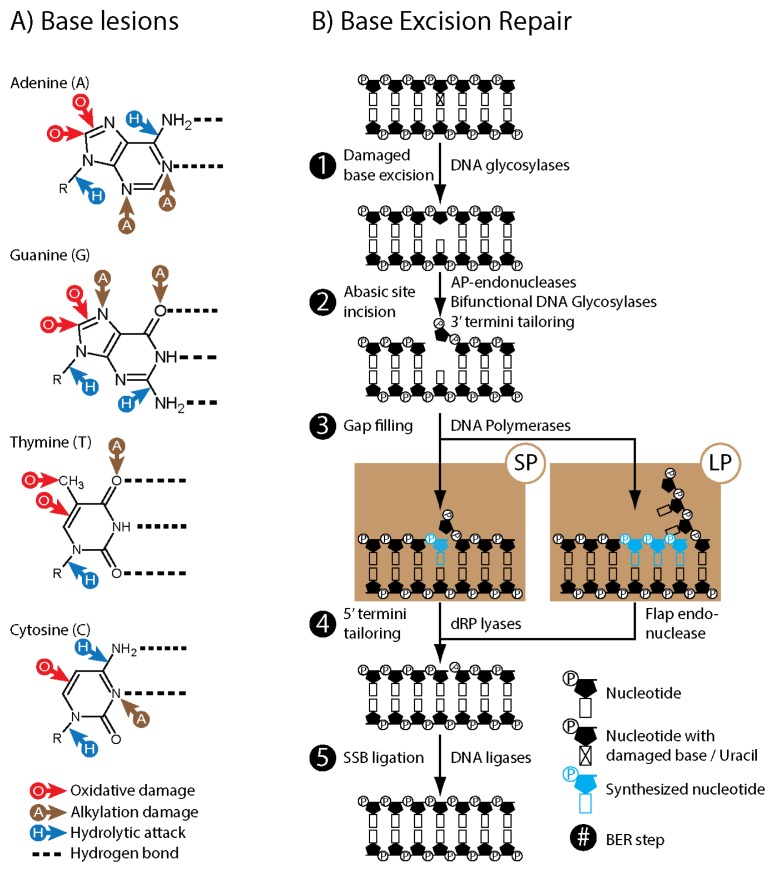
Base lesions and BER summary. (**A**) Base lesions: Typical sites of oxidation (red arrow marked O), alkylation (brown arrow marked (**A**) and spontaneous hydrolysis (blue arrow marked H) within adenine, guanine, thymine and cytosine; (**B**) Base Excision Repair: 1 to 5 major steps and enzymes/enzymatic activities.

DNA base degradation occurs spontaneously through hydrolysis as well as through interaction with reactive molecules. Endogenous alkylating agents such as *S*-Adenosyl methionine and exogenous agents such as chemotherapeutic agents and cigarette smoke metabolites add methyl groups to the nitrogens and oxygens of purines and pyrimidines (Figure 1; panel A). Reactive oxygen species (ROS) generated, e.g., endogenously by the electron transport chain in mitochondria, or by ionizing and UV irradiation, typically add hydroxyl groups to double bonded carbons within purines and pyrimidines, as well as the sugar-phosphate DNA backbone causing strand breaks (Figure 1; panel A). Loss of bases and base lesions that alter base pairing properties of DNA are potentially mutagenic and toxic, and merely spontaneous hydrolysis of bases in DNA is estimated to happen more than 18,000 times per cell per day [11]. Despite this high number of DNA damage, the rate of somatic mutations in humans has been estimated to be 10^−6^ per cell division [12]. The majority of the damaged bases are removed and replaced through BER. Although BER comprises several sub-pathways, it can be generally summarized as five enzymatic steps (Figure 1; panel B). First the *N*-glycosidic bond of the damaged base is cleaved by a DNA glycosylase leaving an abasic site in the DNA. The sugar-phosphate backbone of the abasic site is then cleaved by a bi-functional glycosylase and/or an AP-endonuclease. If necessary the 3’ strand break end is converted to hydroxyl allowing DNA polymerases to reinsert new bases. Synthesis of a single base is referred to as short patch (SP) BER and synthesis of several bases is referred to as long patch (LP) BER. The 5’ single strand end of the single strand break intermediate is then processed to allow for ligation by DNA ligases. In addition to base damage, the BER pathway is also involved in the repair of abasic sites and single strand breaks that are generated independently of glycosylases and/or AP-endonucleases, such as DNA sugar-phosphate backbone cleavage by ROS or spontaneous hydrolysis of the *N*-glycosidic bond.

## 3. XRCC1 Structure and Protein Interacting Regions

**Figure 2:**
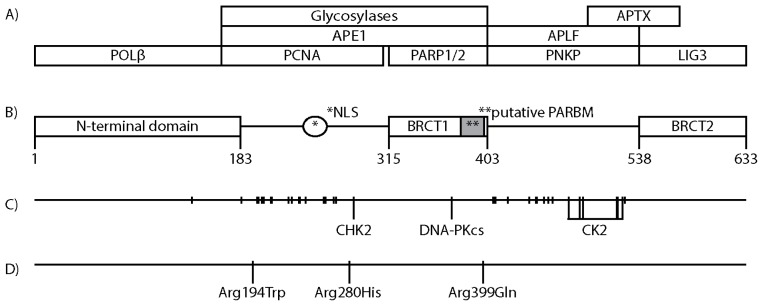
XRCC1 map. (**A**) Approximate XRCC1 protein interacting regions; (**B**) XRCC1 domains; (**C**) Known XRCC1 Ser/Thr phosphorylations and XRCC1 interacting kinases (Chk2: Thr284, DNA-PKcs: Ser371, CK2: cluster of six residues from Ser475 to Ser523, possibly more); (**D**) The three most prevalent XRCC1 variants.

The human *Xrcc1* gene is 33 kilobases long and located on chromosome 19q13.3–13.3 (Ensembl ID: ENSG00000073050). Its product XRCC1 is a 633 amino acid (aa) long protein of approximately 70 kDa (Uniprot ID: p18887) that is translocated to the nucleus by a nuclear localisation signal (NLS) located between aa 239 and aa 266 (Figure 2; panel B) [13]. XRCC1 is partially excluded from nucleolus and accumulate into discrete foci depending on cell cycle phase and cellular stress [14–16].

The region spanning from aa 1 to aa 183, commonly referred to as *N*-terminal domain (NTD), binds DNA polymerase beta (POL*β*) with high affinity (Figure 2; panels A and B) [17,18]. Nuclear magnetic resonance (NMR) crystallography of the NTD and POL*β* has indicated that they form a sandwich like structure that surrounds gapped DNA, suggesting that the NTD might tether XRCC1 to, and protect BER intermediates [19,20]. However, recent NMR studies suggests that POL*β* interacts with an oxidized form of NTD in a manner that excludes XRCC1 interaction with gapped DNA [21]. The interaction with XRCC1 stabilizes POL*β* by protecting POL*β* from degradation, and is required for POL*β* recruitment to DNA damage [16,22].

XRCC1 lacks proliferating cell nuclear antigen (PCNA) interaction motifs such as the PCNA interacting peptide box or AlkB homologue 2 PCNA-interacting motif [23,24]. However, the region spanning from aa 166 to aa 310 of purified recombinant XRCC1 has been demonstrated to interact with purified recombinant PCNA (Figure 2; panels A and B) [14].

The aa 166 to aa 403 region of XRCC1 interacts with nei endonuclease VIII-like 2 (NIEL2), nth endonuclease III-like 1 (NTH1), 8-oxoguanine DNA glycosylase (OGG1), and Uracil-DNA glycosylase 2 (UNG2) [15,25,26]. Furthermore repair activity assays indicate that N-methylpurine DNA glycosylase (MPG) binds within the aa 1 to aa 406 region of XRCC1, although this was not confirmed by immunoblotting [26]. Together, these results indicate that the aa 166 to aa 403 is a central DNA glycosylase interacting region of XRCC1 (Figure 2; panels A and B). In addition, the aa 166 to aa 403 region of XRCC1 appears to interact with APE1 [25,27]. XRCC1 stimulates the activities of recombinant NTH1, OGG1, MPG and APE1 [25–27].

The XRCC1 protein sequence includes two BRCA 1 *C* terminus (BRCT) domains. One is located centrally, BRCT1 aa 315 to aa 403, and one *C*-terminally, BRCT2 aa 538 to aa 633 (Figure 2; panel B). BRCT domains are predominantly found among proteins involved in DNA damage response (DDR), and appears to act as phosphorylation dependent protein interaction domains [28]. Of the two XRCC1 BRCT domains, BRCT1 is the most evolutionary conserved and is required for efficient DNA repair and proliferation after DNA methylation damage [29,30]. Merely the NLS and BRCT1 domain (aa 166 to 436) is sufficient to improve the repair proficiency of methylation induced damage of XRCC1 deficient EM9 CHO [31]. XRCC1 BRCT1 interacts with the BRCT domain of Poly(ADP-ribosyl) polymerase 1 (PARP1), with Poly(ADP-ribosyl) polymerase 2 (PARP2), and encompasses a putative Poly(ADP-ribose) binding motif (PARBM: aa 379 to aa 400) (Figure 2; panels A and B) [13,32,33]. The BRCT2 domain of XRCC1 binds the BRCT domain of Ligase 3 (LIG3) [34,35] and, similar to XRCC1 interaction with POL*β*, this interaction stabilizes the expression levels of LIG3 (Figure 2; panels A and B) [36]. The XRCC1 BRCT domains also serve as inter-XRCC1 interaction modules, although their respective contribution is still somewhat unclear. Beernink *et al.* reported in 2005 that XRCC1 BRCT1 domains could form a heterotetrameric interaction with PARP1, while BRCT2 domains could dimerize directly [37]. In 2006, Lévy *et al.* reported that XRCC1 dimerization was formed through BRCT1 domains and not BRCT2 [38]. In 2011, Cuneo *et al.* reported a crystallographically resolved structure of a XRCC1/LIG3 tetramer with interaction between the XRCC1 BRCT2 domains [39]. Although the details on how XRCC1 forms multimers are diverging, interaction between XRCC1 deletion mutants containing only BRCT1 as well as both BRCT domains have been confirmed *in vivo* by fluorescence resonsance energy transfer (FRET) [14,31].

The region between the two XRCC1 BRCT domains (aa 403 to aa 538) interacts with the forkhead-associated (FHA) domains of aprataxin (APTX), polynucleotide kinase/phosphatase (PNKP), and aprataxin and PNKP like factor (APLF) (Figure 2; panels A and B) [40–45]. FHA domains are involved in protein/protein interactions through phospho-threonine binding, and are found in more than 700 eukaryotic proteins such as kinases, phosphatases, kinesins, transcription factors, RNA binding proteins and metabolic enzymes [46]. Interaction with XRCC1 stimulates both the phosphatase and kinase activities of recombinant PNKP [47].

## 4. Posttranslational Modifications of XRCC1

The most prominent posttranslational modifications (PTM) of XRCC1, and BER proteins in general, known to date are phosphorylations [48]. XRCC1 is a heavily phosphorylated protein with more than 30 phosphorylated Ser/Thr residues (PhosphoSitePlus database, search term “XRCC1”; p18887. Last accessed 31th October 2012. http://www.phosphosite.org) (Figure 2; panel C). Ser371 within BRCT1 has been shown to be phosphorylated *in vivo* by DNA-PKcs upon ionizing irradiation induced DNA damage, and to cause XRCC1 dimer dissociation *in vitro*[38]. However, all other verified phosphorylations of XRCC1 cluster outside of the BRCT domains, mainly within the regions between the NTD and BRCT1 (aa 183 to aa 315), and in the inter BRCT region (aa 403 to aa 538). Checkpoint kinase 2 (CHK2) complexes with XRCC1 and phosphorylates Thr284. XRCC1 mutated at Thr284 was suggested to be linked to accumulation of BER intermediates possibly through modification of XRCC1s interaction with DNA glycosylases [49]. Of the known kinase interactions with XRCC1, casein kinase 2 (CK2) is the most extensively documented. CK2 is a pleiotropic, ubiquitous, and constitutively active kinase. CK2 phosphorylates hundreds of different substrates and among them several factors known to be associated with cellular stress responses, cell proliferation, and cancerogenesis, such as nuclear factor kappa light chain enhancer of activated B cells (NF-kappaB). CK2 expression is up regulated in a wide variety of human cancers and has become an interesting target for drug design [50]. The region between the BRCT domains of XRCC1 encompasses eight primary and five atypical consensus sites for CK2, and they are readily phosphorylated by CK2 *in vitro*[42]. Nine of the Ser/Thr residues were observed to be phosphorylated *in vivo*, and among them six residues close to BRCT2 showed reduced phosphorylation when CK2 was knocked down (Figure 2; panel C) [42,43]. Phosphorylations of residues in the inter BRCT region stimulate binding of XRCC1 to the FHA domains of APTX, PNKP, APLF [40–45].

## 5. XRCC1 Multiprotein Complexes

**Figure 3:**
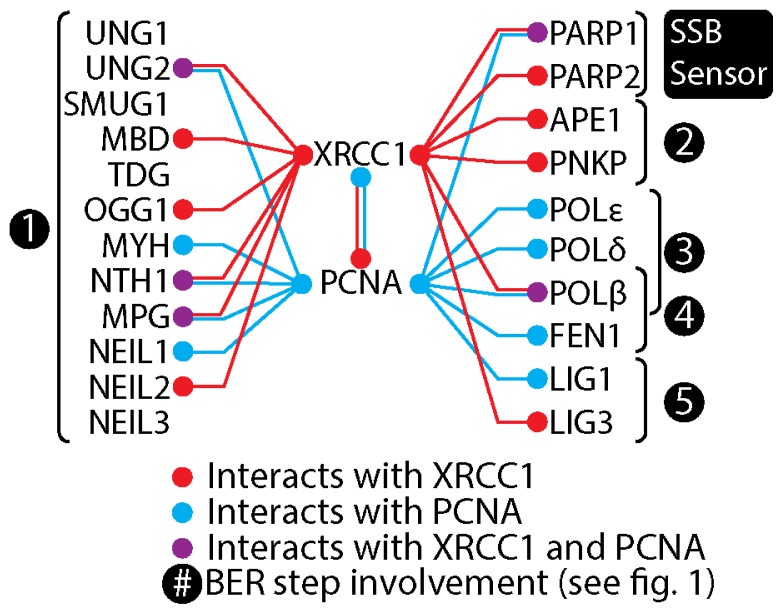
Scaffold protein interactions in BER. Common and separate interactions of XRCC1 and PCNA with enzymes involved in BER.

With the exception of POL*β* and LIG3, the remaining XRCC1 binding proteins show extensive overlap in their binding epitopes. Furthermore, only the POL*β* and LIG3 interactions are considered strong. This has lead to the notion that XRCC1 contributes to BER as a co-factor or temporary docking platform through a succession of interactions and enzymatic events. However, the dimeric interactions between XRCC1 BRCT domains and heterotetrameric assembly of XRCC1 with either PARP1 or LIG3 observed *in vitro* as well as by FRET suggest that XRCC1 complexes may be able to contain many factors [14,37–39]. The assembly of large multiprotein complexes is plausible because many of the XRCC1 interacting proteins have been shown to interact among themselves (reviewed in [48]). In addition, XRCC1 interacts with PCNA, a well-known scaffolding protein (Figure 3).

In 2004 results from our group indicated the presence of large BER proficient multiprotein complexes as UNG2 co-immunoprecipitated with PCNA, XRCC1, and other proteins that bind both XRCC1 and PCNA [51]. Similarly Heale *et al.*, and Parlanti *et al.* detected XRCC1 in multiprotein complexes associated with Condensin 1 and Cyclin A, in addition to proteins that are not known to interact directly with XRCC1, (e.g., POL*δ*, POL*ε*, and FEN1) [52,53]. However, co-immunoprecipitation results only suggest the presence of multiprotein complexes as protein interactions may form after cell lysis. The presence of XRCC1 associated multiprotein complexes containing proteins that have overlapping interaction regions within XRCC1 (PCNA and PARP1 *vs*. UNG2 and APE1) were confirmed by mild *in vivo* formaldehyde crosslinking prior to co-immunoprecipitation studies. Furthermore, proteins with no known interaction with XRCC1 (POL*δ* and FEN1) were also found in these complexes [15]. Gel fractionation of cell extracts confirmed the presence of large XRCC1 complexes of various compositions and sizes ranging from 150 to 1500 kDa. The highest BER activity was found in complexes isolated from fractionated extracts ranging in size from 350 to 700 kDa [16]. *In vitro* studies of interactions between BER enzymes and their DNA substrates indicate that the enzymes stay bound to their product, preventing genotoxic and mutagenic effects of repair intermediates before handing them over to the next enzyme [54]. Multiprotein BER complexes could contribute to tethering of factors to the lesions and repair intermediates and thus provide a platform for coordinated progression of repair, and also increase the rate of repair.

## 6. The Composition of XRCC1 Associated Multiprotein Complexes

The involvement of replication factors such as POL*δ*, POL*ε*, FEN1, and PCNA in LP BER, and the deleterious effects of replication past SSBs and certain base lesions have long suggested the presence of replication-coupled BER/SSBR [17,55,56]. The presence of XRCC1 in replication foci and its co-immunoprecipitation with Cyclin A associated proteins further suggest a role for XRCC1 in replication associated repair [14,53]. More recent co-immunoprecipitation data from XRCC1 associated and UNG2 associated complexes isolated from S-phase enriched cells, demonstrated that these complexes contained both common and separate factors. Colocalization studies showed that XRCC1, PNKP and POl*β* were found both within and outside of replication foci, while UNG2 was only found in association with the replication machinery. Additionally, the different complexes showed different BER proficiencies. XRCC1-associated complexes were far more efficient in BER than UNG2-associated complexes, indicating the presence of distinct BER complexes during S-phase [15,16].

**Figure 4:**
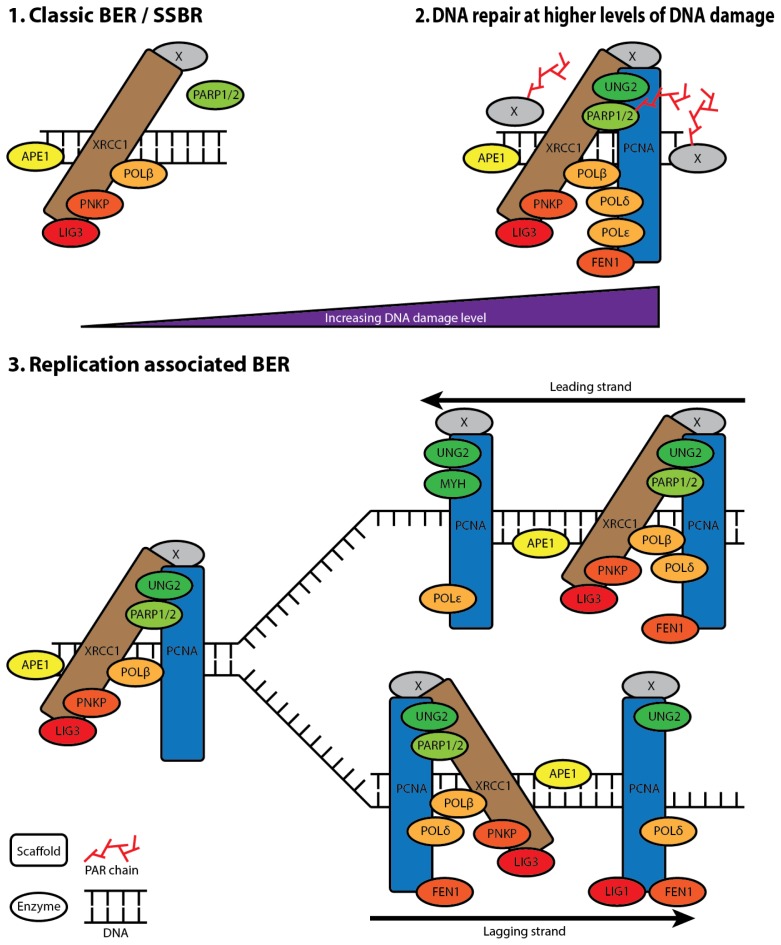
A model of BER multiprotein complexes. (**1**) Classic BER/SSBR: At endogenous or low levels of induced DNA damage. Not dependent upon PARP1 and PARylation. Represents XRCC1-foci observed in untreated cells. Contain XRCC1 core-complex, APE1 and PARPs. Other proteins (X); (**2**) DNA repair at higher levels of DNA damage: Higher levels of DNA damage including strand breaks recruits PARPs followed by extensive PARylation. This is important both for signalling of damage, recruitment of repair factors and chromatin remodelling, and therefore the repair rate. These XRCC1-foci contain in addition to the core-complex, DNA repair proteins known to be involved replication and LP BER such as POL*δ*, FEN-1, PCNA and more (X); (**3**) Replication associated BER: Pre-replicative repair of single strand breaks and base damages prior to replication likely includes the UNG2 DNA glycosylase, and possibly MPG, in addition to the core-complex. Rapid removal of misincorporated bases by the DNA glycosylases UNG2 and MYH prior to rapid repair of abasic sites by the XRCC1 core complex on both leading and lagging strand. However, presence of FEN1, POL*δ*, and LIG1 on the lagging strand possibly also enables LP BER by “UNG2 associated complexes” [15]. This figure is adapted from [16].

We have found that the repertoire of proteins recruited to near-UVA (405 nm) induced DNA damage is highly dependent on the radiation dose. Low doses result in recruitment of BER factors that are also observed in foci independent of exogenously induced DNA damage, e.g., PNKP, and POL*β*, while high doses in addition resulted in the recruitment of PCNA and FEN1 [16]. In sum, these observations suggest the presence of a “XRCC1 core complex” associated with the replication machinery as well as sites of endogenously and exogenously induced DNA damage (Figure 4).

The strong interaction between XRCC1 and LIG3 and the observed AP-endonuclease activity of low mass XRCC1 associated complexes indicate that the core complex in addition to PNKP and POL*β* also contains LIG3 and APE1 [15,16,57,58]. The core complex is extended to include proteins involved in replication, LP BER, and other repair pathways when levels of damage reach a certain threshold. However, while the presence of XRCC1 reduces the near-UVA micro-irradiation doses required for PCNA recruitment, PCNA is recruited to DNA damage in XRCC1 deficient EM9 CHO cells, indicating the involvement of PCNA in DDRs that are independent of XRCC1 [16,31].

PCNA is a central scaffolding protein in the replication machinery as well as in LP BER, and several BER proteins have been demonstrated to co-immunoprecipitate with PCNA (Figure 3) [14,56,59–69]. Interestingly, while the aa 166 to aa 310 region of XRCC1 is required for co-immunoprecipitation of XRCC1 and PCNA, it is not required for colocalization of XRCC1 with PCNA in replication foci during S-phase. The colocalization with PCNA in replication foci requires the aa 310 to aa 436 region, which includes the BRCT1 domain, suggesting that XRCC1 may be recruited to the replication foci via interaction with proteins other than PCNA [14,31]. PARP1, MPG, and UNG2 are all found in replication foci, interact with PCNA, and within or partially with the aa 310 to aa 436 region of XRCC1 [13,15,26,56,66,70].

## 7. XRCC1 Recruitment to DNA Damage

To date, eleven DNA glycosylases have been identified in humans [71] (The URL cited in this article is erroneous. An updated table based on this article is available at the following URL, last accessed 18th October 2012. http://sciencepark.mdanderson.org/labs/wood/DNARepairGenes.html#HumanDNARepairGenes). Although the overall folding architectures of DNA glycosylases are similar, their substrate specificities are generally different. How DNA glycosylases are able to detect or get recruited to the plethora of different damaged bases buried within the DNA double helix and chromatin remains elusive, but it involves association to replication, and possibly transcription [72]. We have not been able to induce intracellular re-location of UNG2, MPG or OGG1 to near-UVA micro-irradiated regions, or detect their presence in constitutively present XRCC1 foci, suggesting that these glycosylases are not recruited together with XRCC1. However, this is difficult to assess as most methods for generation of base lesion produce a variety of different lesions that are substrates to different DNA glycosylases, as well as strand breaks that are sensed by PARPs.

Recruitment of XRCC1 to near-UVA micro-irradiated regions of the nucleus is strongly influenced by the aa 166 to aa 310 region and depends on the presence of the BRCT1 domain (aa 315 to aa 403) [31]. While DNA glycosylases interact with both regions, PARP1 and PARP2 interact with XRCC1 within the BRCT1 domain [13,33]. PARP1 and PARP2 bind to and get activated by SSBs [73,74]. Upon activation, PARP1 and PARP2 synthesize branched chains of up to 200 ADP-ribose moieties on themselves and many other target proteins. Poly(ADP)ribose (PAR) chains are rapidly disassembled by PAR glycohydrolase, making PARylation a transient event that last only minutes [75]. The ADP-ribose units are negatively charged and PAR chains thus potentially cause both steric and charge interactions with other molecules [76]. PARylation of histones in response to DNA damage contributes to chromatin reorganization, and PAR chains mediate signalling of the genomic state and serves as recruitment platforms for other proteins [77]. Three PAR association motifs have been described: a macrodomain, a PAR-binding Zinc finger motif, and a cluster of eight amino acids rich in acidic and hydrophobic residues (PARBM). Together these PAR association domains interact with more than 300 proteins, among which DDR proteins are overrepresented in addition to many DNA replication and transcription factors [78]. In addition to the reported PARP1 and PARP2 interaction, a putative PARBM have been identified within the XRCC1 BRCT1 domain, making both PARP1 and PARP2, and PAR chains plausible candidates as initiators of XRCC1 complex recruitment to SSB [13,32]. In 2003 El-Khamisy *et al.* reported abrogation of XRCC1 foci formation by inhibition of PARylation, and foci reduction by point mutation within the putative PARBM in BRCT1, suggesting that XRCC1 recruitment to SSB is primarily mediated through interaction with PAR chains rather than PARP1 [79]. This was later supported by Mortusewicz *et al.* and Godon *et al.* who observed strong reduction of XRCC1 recruitment and increased PARP1 accumulation in near-UVA microirradiation induced foci by inactivation of PARP1s PARylation domain or by use of PARylation inhibitor 4-AN (4-amino-1, 8-naphtalimide; CAS: 1742-95-6 ) [80,81]. However, in our hands treatment with 4-AN or the structurally unrelated PARylation inhibitor PJ34 (CAS: 344458-15-7) caused only minor reduction in XRCC1 recruitment to low dose near-UVA micro-irradiated regions of the nucleus [16]. While XRCC1 accumulation did not coencide with increased accumulation of non-PARylated PARP1, confirming that XRCC1 recruitment is independent of direct interaction with non-PARylated PARP1, they do not support the notion that PARylation activity or PAR chains are required for XRCC1 recruitment to SSBs. The slight reduction of XRCC1 recruitment upon 4-AN and PJ34 treatment might rather be mediated by indirect mechanisms such as PARylation mediated chromatin reorganization.

## 8. XRCC1 Variants

XRCC1’s role as a scaffolding protein in BER and its observed effect on the activity of certain BER factors have prompted a number of studies assessing the influence of the three most prevalent XRCC1 polymorphisms Arg194Trp (rs1799782), Arg280His (rs25489), and Arg399Gln (rs25487) on genomic stability (Figure 2; panel D) (summary available in [82]). Studies of primary cells from individuals carrying XRCC1 polymorphisms commonly involve DNA damage induction in heterozygous lymphocytes by alkylating-, oxidizing-, strand break inducing- agents or by ionizing radiation followed by measurement of markers of genome instability such as SCE and micronuclei formation. These studies are limited by sample sizes, and therefore difficult to interpret. Most agents used to induce base lesions also produce lesions or lesion clusters that involve other DNA repair pathways such as NER, trans-lesion synthesis, and double strand break repair (DSBR). Furthermore, dysregulation of any DNA repair mechanism that resolve or intermediately produce strand breaks can be associated with genomic fragmentation and thus influence the studied markers of genomic instability. However, the studies in sum suggest that the Arg194Trp variant is associated with increased genomic stability in response to DNA damaging agents, whereas the Arg280His and Arg399Gln variants are associated with reduced genomic stability (details are available in Table 1 of [82]). Altered BER efficiency caused by XRCC1 polymorphisms might be mediated by changes in protein-protein interactions, XRCC1 recruitment, or XRCC1 influence on BER enzyme activities. *In vivo* studies of XCC1 deficient EM9 CHO expressing polymorphic XRCC1 constructs demonstrated that Arg280His and Arg399Gln XRCC1, respectively accumulate less into and dissociates faster from near-UVA micro-irradiation induced DNA damage. Single cell gel electrophoresis indicated that these variants had subtle differences in their repair profiles of oxidative damage induced by hydrogen peroxide, supporting that small changes in the scaffolding properties of XRCC1 may affect DNA repair and thus genomic stability [31,83].

## 9. XRCC1 and Disease

Base lesions, abasic sites and single strand breaks are relatively common DNA lesions because they are generated continuously by endogenous reactive agents, erroneous DNA processing/synthesis, as well as spontaneously. Minor changes in BER efficiency that alter genomic stability can potentially have a big impact on cancer risk. The Arg194Trp, Arg280His, and Arg399Gln XRCC1 variants have been extensively studied in relation to cancer. However, metastudies of the epidemiological datasets have so far not yielded any unambiguous relationships to cancer prevalence [84–87]. This is probably a result of both the size of populations included in the specific epidemiological studies and significant differences in the genomic single nucleotide polymorphism (SNP) distribution between studied populations (Table 1). For example, homozygous Arg280His has been observed to be associated with breast cancer in Asians, but not in Caucasians [88]. Homozygous Arg194Trp increases risk of lung cancer in Asians, whereas heterozygous Arg194Trp in Caucasians reduces the risk [89]. However, more data regarding XRCC1 and cancer susceptibility is published weekly, and in a recent meta-analysis the Arg399Gln variant was found to be associated with increased risk of developing breast cancer [90].

DNA synthesis and ligation require 3’ hydroxyl and 5’ phosphate ends. The repair of SSB and BER intermediates typically involve DNA-end processing enzymes, and many have been demonstrated to interact directly or indirectly with XRCC1; e.g., APTX and TDP1, respectively involved in processing 5’ ends produced by abortive ligation, and 3’ ends produced by abortive double stranded unwinding by Topoisomerase 1 [45,91,92]. Mutations in the catalytic domains of TDP1 and APTX are associated with subtypes of the neurodegenerative disease spinocerebellar ataxia [91,93,94]. As previously mentioned, the phosophorylation status of the inter BRCT domain region of XRCC1 (aa 403 to 538) influences the interaction with the FHA domains of PNKP, APTX and APLF. Additionally, PNKP is functionally stimulated by interaction with XRCC1 [47]. XRCC1 thus influences SSB end processing both as an enzymatic co-factor and through its scaffolding properties, and functional changes of XRCC1 could therefore potentially contribute to disease. The observation that conditional neuron disruption of *Xrcc1* in mice influences neuronal development and causes accumulation of DNA strand breaks makes it plausible that XRCC1 variants might contribute to neurodegenerative diseases and influence glioma risk [8]. Recent epidemiological studies suggest that Arg399Gln XRCC1 is associated with Parkinson’s disease, sporadic amyotrophic lateral sclerosis, and increased glioma risk [95–98]. Furthermore, consistent with the general observations, the Arg194Trp variant has been found to be associated with a reduced glioma risk [99]. While XRCC1 polymorphisms probably influence BER/SSBR efficiency, it is important to notice that XRCC1 participation in DNA repair is not limited to BER/SSBR. XRCC1 and XRCC1 interacting factors have been shown to be involved in NHEJ and to be recruited to UVC induced DNA damage during NER [6,7,100]. Whether and how different XRCC1 variants affect NHEJ or NER is not known. XRCC1’s contribution to repair of UV induced DNA damage might be affected by the diverging recruitment kinetics of the XRCC1 variants, and this could influence the risk of skin cancer. In accordance with this hypothesis epidemiological studies of XRCC1 variants have associated Arg399Gln and Arg280His with an increased risk of developing melanoma and Arg399Gln and Arg194Trp with an increased risk of developing squamous cell carcinoma [101,102]. However, as noted by Santonocito *et al.* in 2012, concerning both XRCC1 and OGG1 variants, *“all previous papers seemed to exclude possible association between the (non-synonymous) SNPs considered in our study and (malignant melanoma)”*[102]. Thus larger mulitcenter studies are required.

In sum, because XRCC1 variants may alter XRCC1’s capacity to form multiprotein complexes, influence the enzymatic activity of protein partners and alter the recruitment to DNA damage, they may influence the risk of disease. However, as XRCC1 is an essential protein during foetal development, a strong negative selection against any prominent functional changes of XRCC1 is likely.

## Figures and Tables

**Table 1:** Allele frequencies of the three most prevalent *Xrcc1* SNPs known to date.

*Xrcc1* (ENST00000262887)		Africa		America		Asia		Europe	
aa substitution	SNP ID	Hom.	Het.	Hom.	Het.	Hom.	Het.	Hom.	Het.
Arg194Trp	rs1799782	N.D.	14.6%	0.6%	19.9%	8.4%	40.9%	1.3%	9.0%
Arg280His	rs25489	N.D.	2.8%	0.6%	12.2%	1.0%	16.8%	0.5%	11.6%
Arg399Gln	rs25487	0.8%	22.0%	9.4%	43.1%	6.6%	36.7%	11.9%	45.4%

Hom.: homozygous, Het.: heterozygous. Data from the ENSEMBL database. Last accessed 29th November 2012. http://www.ensembl.org.
